# Deriving Freshwater Quality Criteria for Iron, Lead, Nickel, and Zinc for Protection of Aquatic Life in Malaysia

**DOI:** 10.1100/2012/861576

**Published:** 2012-08-02

**Authors:** M. Shuhaimi-Othman, Y. Nadzifah, R. Nur-Amalina, N. S. Umirah

**Affiliations:** School of Environmental and Natural Resource Sciences, Faculty of Science and Technology, Universiti Kebangsaan Malaysia (UKM), Selangor, 43600 Bangi, Malaysia

## Abstract

Freshwater quality criteria for iron (Fe), lead (Pb), nickel (Ni), and zinc (Zn) were developed with particular reference to aquatic biota in Malaysia, and based on USEPA's guidelines. Acute toxicity tests were performed on eight different freshwater domestic species in Malaysia which were *Macrobrachium lanchesteri* (prawn), two fish: *Poecilia reticulata* and *Rasbora sumatrana, Melanoides tuberculata* (snail), *Stenocypris major* (ostracod), *Chironomus javanus* (midge larvae), *Nais elinguis* (annelid), and *Duttaphrynus melanostictus* (tadpole) to determine 96 h LC_50_ values for Fe, Pb, Ni, and Zn. The final acute value (FAV) for Fe, Pb, Ni, and Zn were 74.5, 17.0, 165, and 304.9 **μ**g L^−1^, respectively. Using an estimated acute-to-chronic ratio (ACR) of 8.3, the value for final chronic value (FCV) was derived. Based on FAV and FCV, a criterion maximum concentration (CMC) and a criterion continuous concentration (CCC) for Fe, Pb, Ni, and Zn that are 37.2, 8.5, 82.5, and 152.4 **μ**g L^−1^ and 9.0, 2.0, 19.9, and 36.7 **μ**g L^−1^, respectively, were derived. The results of this study provide useful data for deriving national or local water quality criteria for Fe, Pb, Ni, and Zn based on aquatic biota in Malaysia. Based on LC_50_ values, this study indicated that *N. elinguis, M. lanchesteri, N. elinguis,* and *R. sumatrana* were the most sensitive to Fe, Pb, Ni, and Zn, respectively.

## 1. Introduction

Metal contamination has been shown to have serious effects on both the environment and humans. Malaysia, as a developing country, is no exception and faces metal pollution caused especially by anthropogenic activities such as manufacturing, agriculture, sewage, and motor vehicle emissions [1, 2]. Studies on metals in water and sediments indicate that some rivers in Malaysia were contaminated with As, Ag, Cd, Cu, Pb, and Zn and some coastal sediments were contaminated by Pb, Zn, and Cd [1–4]. However, Malaysia has a lack of water quality criteria (WQC) based on local aquatic biota. The existing water quality standards (WQSs) for metals in Malaysia (National Water Quality Standards) are based mainly on foreign criteria or standards, which have different environmental conditions compared to Malaysia. Many factors (physical, chemical, and biological) are known to affect the toxicity of metals to aquatic organisms. These factors, especially the differences in taxonomic composition of Malaysian waters compared to those for which WQSs were developed, could result in foreign water quality criteria or standards that are overprotective or underprotective for aquatic ecosystems in Malaysia. In order to protect aquatic ecosystems in Malaysia, it is necessary to develop WQC for metals based on the responses of domestic aquatic biota with local environmental factors. This information could also be used to determine sensitive and potential organisms as bioindicator for metal pollution especially in Malaysia.

Metals such as Fe, Pb, Ni, and Zn are released from natural sources as well as human activity. Despite the adverse effects of metals on the environment and organisms, some metals are essential to living organisms. Zn plays an important role as a prosthetic group for the enzyme carbonic anhydrase while Fe in the respiratory protein haemoglobin [5]. Toxicity testing is an essential tool for assessing the effect and fate of toxicants in aquatic ecosystems and has been widely used as a tool to identify suitable organisms as a bioindicator and to derive water quality standards for chemicals [6]. Macroinvertebrate as a test organisms in toxicity tests have several valuable characteristics such as their widespread distribution and common occurrence in freshwater, their ecological importance and ease of handling during testing, as well as their rapid growth, short life cycle and sensitivity to contaminants [7, 8]. Therefore, these organisms have the potential to act as a bioindicator of heavy metals pollution in an aquatic environment and as organisms for toxicity testing. USEPA [9] produced technical guidelines to give an objective way of deriving numerical national WQC. Acute to chronic ratios (ACRs) have been used extensively in ecological risk assessment to estimate the chronic toxicity of chemicals in aquatic organisms for which acute toxicity is known but data regarding chronic toxicity are either limited or absent. The “final acute value” (FAV) is often divided by an acute-to-chronic toxicity ratio (ACR) to estimate a chronic criterion that would not result in unacceptable adverse effects to aquatic communities. Although the ACR approach has weaknesses for criteria development or risk assessment, a major strength of the ACR approach is that it allows estimates of chronic values for acutely sensitive species to be made for which no chronic data are available. In such cases, direct analysis of available chronic data may underestimate chronic toxicity, whereas the ACR allows some extrapolation of chronic effects for sensitive species, even though no chronic data exists [10].

In this study, freshwater WQCs were developed for metals (Fe, Pb, Ni, and Zn) based on its acute toxicity to freshwater fish and invertebrates using domestic aquatic organisms. The toxicity data for Fe, Pb, Ni, and Zn were generated by conducting acute toxicity testing with eight fish and invertebrate species and the Criterion Maximum Concentration (CMC) was derived. An estimated value of chronic data using acute-to-chronic ratio (ACR) was used to derive the Criterion Continuous Concentration (CCC). The overall objective of this study was to provide useful data to derive national or local water quality criteria for Fe, Pb, Ni, and Zn based on aquatic biota indigenous in Malaysia.

## 2. Materials and Methods

### 2.1. Organisms and Test Chemicals

In this study eight local freshwater organisms have been used in toxicity testing, that is, a prawn *Macrobrachium lanchesteri*, two fish: *Poecilia reticulata* (guppy, family Poecilidae) and *Rasbora sumatrana* (family Cyprinidae), a snail (Gastropoda) *Melanoides tuberculata *(family Thiaridae), an ostracod *Stenocypris major*, a midge larvae *Chironomus javanus* (Diptera, Chironomidae), an annelid *Nais elinguis*, and a tadpole *Duttaphrynus melanostictus*. *M. lanchesteri* and *R. sumatrana* were obtained from local pet stores. *P. reticulata, D. melanostictus*, and *M. tuberculata* were collected from the field. *N. elinguis*, *S. major*, and *C. javanus* were collected from a fish pond filter system. Prior to toxicity testing, the organisms were acclimatized for one week under laboratory conditions (28–30°C with 12 h light : 12 h darkness) in 20-L stocking tanks using dechlorinated tap water (filtered by several layer of sand and activated carbon; T.C. Sediment Filter (TK Multitrade, Seri Kembangan, Malaysia)) and aerated through an air stone. During acclimation the organisms were fed with commercial fish food Tetramin (Tetrawerke, Germany). Four metals were used in this toxicity testing which were iron (Fe), lead (Pb), nickel (Ni), and zinc (Zn). The standard stock solution (100 mg L^−1^) of Fe, Pb, Ni, and Zn were prepared from analytical grade metallic salts of FeCl_3_, Pb(NO_3_)_2_, NiSO_4_·6H_2_O, and ZnSO_4_·7H_2_O (Merck, Darmstadt, Germany). The stock solutions were prepared with deionized water in 1-L volumetric.

### 2.2. Acute Toxicity Test

Acute Fe, Pb, Ni, and Zn toxicity experiments were performed for a four-day period (96 h) using adult animals or larvae (fourth instar midge larvae and tadpole). Following a range finding test, five Fe, Pb, Ni, and Zn concentrations were chosen. Metal solutions were prepared by dilution of a stock solution with dechlorinated tap water. A control with dechlorinated tap water only was also used. The tests were carried out under static conditions with renewal of the solution every two days. Control and metal-treated groups each consisted of two to four replicates of five randomly allocated organisms. No significant stress was observed for the organisms in the solution indicated by 95–100% survival for the organism in the control water until the end of the study. For each species, a total of 10 to 20 animals per treatment (concentrations) were used in the experiment. Samples of water for metal analysis taken before and immediately after each solution renewal were acidified to 1% with ARISTAR nitric acid (65%) (BDH Inc., VWR International Ltd., England) before metal analysis by flame or furnace Atomic Absorption Spectrophotometer (AAS–Perkin Elmer (MA, USA), model Analyst 800) depending on the concentrations. To avoid possible contamination, all glassware and equipment used were acid-washed (20% HNO_3_ (Dongbu Hitek Co. Ltd., Seoul, Korea, 68%)) and the accuracy of the analyses was checked against blanks. Procedural blanks and quality control samples made from standard solution for Cu, Cd, Al, and Mn (Spectrosol, BDH, England) were analysed in every ten samples in order to check for samples accuracy. Percentage recoveries for metals analyses were between 85–105%. Details of the experiments can be found in Shuhaimi-Othman et al. [11–16].

During the toxicity test, organisms were not fed. The experiments were performed at room temperature of 28–30°C with photoperiod 12 h light : 12 h darkness, using fluorescent lights (334–376 lux). Water quality parameters (pH, conductivity, and dissolved oxygen) were measured every two days using portable meters (model Hydrolab Quanta, Hach, Loveland, USA) and water hardness samples were fixed with ARISTAR nitric acid and measured by flame atomic absorption spectrophotometer (AAS–Perkin Elmer Analyst 800). Mortality was recorded every 3 to 4 hours for the first two days and then at 12 to 24 hour intervals throughout the test period. Any dead animals were removed immediately.

### 2.3. Statistical Analyses and Data Intergration

Median lethal concentrations (LC_50_) for the animals exposed to Fe, Pb, Ni, and Zn were calculated using measured metal concentrations. FORTRAN programs based on the methods of Litchfield [17] and Litchfield and Wilcoxon [18] were used to compute the LC_50_. Interpretation of toxicity data was conducted according to the methods described in the guidelines of USEPA [9]. Final acute value (FAV) was derived using the FAV equation in the guidelines. The criteria maximum concentration (CMC) was the FAV divided by two. To obtain the final chronic value (FCV), the FAV was divided by the ACR. ACRs have been used to estimate chronic toxicity for chemicals and species with known acute toxicity but limited or no information regarding chronic toxicity. In this study because no chronic data are available for species used in the acute toxicity study, an overall median value of 8.3 for the ACR was used, based on a study by Raimondo et al. [19] who derived a median value for ACR based on 456 same-species (invertebrate and fish) pairs of acute and maximum acceptable toxicant concentrations for metals, narcotics, pesticides, and other organic chemicals. The value of FCV was considered as the criterion continuous concentration (CCC).

## 3. Results and Discussion

The mean water quality parameters measured during the test were pH 6.68 ± 0.2, conductivity 180.3 ± 4.6 *μ*S cm^−1^, dissolved oxygen 6.25 ± 0.3 mg L^−1^, and total hardness (Mg^2+^ and Ca^2+^) 18.72 ± 1.72 mg L^−1^ as CaCO_3_. Results of acute toxicity tests using eight aquatic species (Table 1) showed that *N. elinguis*,* M. lanchesteri*,* N. elinguis*, and* R. sumatrana *were the most sensitive species to Fe, Pb, Ni, and Zn, respectively, while *M. tuberculata, S. major*, and* C. javanus* were the most resistant. Snail *M. tuberculata* was the most resistant to Fe and Pb. According to Von Der Ohe and Liess [20] 13 taxa belonging to Crustacea were among the most sensitive to metal compounds, and they concluded that taxa belonging to Crustacea are similar to one another and to *Daphnia magna* in terms of sensitivity to organics and metals and that Molluscs have an average sensitivity to metals. Mitchell et al. [21] reported that the snail has a tightly sealing operculum that allows it to withstand desiccation and apparently also increases its tolerances to chemicals. Brix et al. [22] also reported that warm water fish, crustaceans other than cladocerans and other invertebrates were consistently of intermediate sensitivity and insects were the least sensitive taxonomic group evaluated for five metals (Cd, Cu, Pb, Ni, Zn). *N. elinguis* is a freshwater worm from Naididae family and a cosmopolitan species that is abundant in organically enriched sites. *N. elinguis* was also reported to be the dominated worm in the activated sludge tank and sewage filter beds [23, 24]. Chapman et al. [25] suggested that metal tolerances in aquatic oligochaetes are species-specific and worm tolerance to Cd and Hg were reverse of sewage sludge tolerances. Fish *R. sumatrana* was also found to be sensitive to Zn. Zakaria-Ismail and Fatimah [26] reported on the tolerance levels of common freshwater fish in Peninsular Malaysia and concluded that *R. sumatrana *has a medium tolerance level to pollutants with a value of 2.5 (value ranged from 0.5 being the most sensitive to 4.5 being the most tolerant species). 

Comparison of toxicity between organisms in rank 1 (most sensitive) and 8 (most resistant) showed that for Fe, Pb, Ni, and Zn, the toxicity was 71, 195, 31, and 12 times lower, respectively (Table 1). Differences in sensitivity for Pb were highest among the four metals studied. The difference seen for trace metals might be explained by metallothionein (MT) synthesis, which is believed to play a protective role against toxic metals in aquatic animals [27, 28]. Other studies also provided evidence that the hypothalamic-pituitary-adrenocortical (HPA) axis, crucial in vertebrates coping with stressors, is one of the metal targets in several animal species, including teleost fish [29, 30]. According to Luoma and Rainbow [5], the rank order of toxicity of metals will vary among organisms, and the factors that affect the rate of uptake of metals affect the toxicity of metal. Metal toxicity results when metals accumulate at an undesirable site(s) in the organisms and disrupt important molecular functions. Toxicity ensues once the threshold of metal availability has been passed, indicating that the rate of uptake exceeds both the rate of excretion and detoxification. Metals also can inhibit the uptake of major ions (Na^+^, Ca^2+^, Mg^2+^, Cl^−^) by freshwater organisms through either competitive or direct inhibition [31].

Using the FAV equation of USEPA [9], a final acute value (FAV) for Fe, Pb, Ni, and Zn of 74.5, 17, 165, and 304.9 *μ*g L^−1^, respectively, was estimated. A criterion maximum concentration (CMC) for Fe, Pb, Ni, and Zn of 37.2, 8.5, 82.5, and 152.4 *μ*g L^−1^, respectively, was obtained by dividing FAV values by 2 (Table 2). A criterion continuous concentration (CCC) was derived by dividing FAV values by ACR. In this study an ACR value of 8.3 was used based on the study by Raimondo et al. [19]. Based on the FAV and ACR values, a criterion continuous concentration (CCC) for Fe, Pb, Ni, and Zn of 9.0, 2.0, 19.9, and 36.7 *μ*g L^−1^, respectively, was obtained (Table 2). Comparison with other WQC or standards for metal from other countries is shown in Table 3. Results of this study were comparable with metal criteria from other countries such as the United States [32], Europe [33], and Canada [34]. However, USEPA standards for Pb which have been adjusted for water hardness 20 mg L^−1^ are lower than the present study. In comparison with current Malaysia water quality standards (National Water Quality Standard) NWQS, [2], values for the standard only given for Class II (clean) and no values for Class I (very clean) were given (only natural level are stated). Therefore the CCC and CMC values derived from this study are suggested to be used in the NWQS Malaysia.

In the present study, water hardness used was considered low (18.7 mg L^−1^ CaCO_3_), and the water was categorized as soft water (<75 mg L^−1^ as CaCO_3_). Low water hardness has been known to increase metal toxicity to organisms [35–38]. This variance in toxicity is primarily the result of cations (Ca^2+^, Mg^+^) competing with metal ions for active binding sites with metal ions thereby limiting metal bioavailability. Most of the Malaysian freshwater ecosystem has low water hardness and normally less than 30 mg/L CaCO_3_ such as freshwater lakes, Lake Chini with hardness <10 mg L^−1^ [39], Lake Bera with hardness 5.4 mg L^−1^ [40], and Lake Bukit Merah with hardness 5 mg L^−1^ [41], and rivers such as Bebar River with hardness <10 mg L^−1^ [42], Kelantan River with hardness 16.1 mg L^−1^ [43] and Langat River with hardness 23.4 mg L^−1^ [44], and this has made the organisms sensitive to metal pollution. In addition, a comparison of metal concentrations in some rivers of Malaysia, such as Langat, Gombak, Mamut, and Linggi rivers, showed the Pb and Zn concentrations to be between 22–75 *μ*g L^−1^, 10–42 *μ*g L^−1^, 2–22 *μ*g L^−1^ and 0.28–0.84 mg L^−1^, respectively [45–48], which were higher than the CMC or CCC values derived from this study. Therefore, the high metal concentration in the water and the low water hardness has made the Malaysian freshwater ecosystem vulnerable and sensitive to metal contamination.

A comparison on toxicity of metals to freshwater organisms revealed that among the four metals studied, Pb was the most toxic to the organisms followed by Fe, Ni, and Zn. Based on other international standards (Table 3), all the standards (USEPA, CCME, and UNECE) categories of Pb were the most toxic to freshwater organisms among the four metals tested. Many studies also showed that Pb was more toxic than Zn, Ni, or Fe to freshwater organisms such as the worm *Lumbriculus variegatus* [49] and *Tubifex tubifex* [50], midge larvae* Chironomus riparius* [51] and *Chironomus tendipes* [52]. Khangarot [50] explained that most of the heavy metal ions are toxic to living organisms because they combine with some ligands of enzymes which are necessary for life. However, for nontransitional metal cation, enzyme inhibition is not likely to be a primary cause of toxicity. But osmotic or other colligative factors working through physical reactions cause physical damage to the cellular system.

The CMC and CCC values of Fe, Pb, Ni, and Zn obtained in our study will provide useful data from which national and local WQC for metals can be derived. The guidelines were developed on the theory that effects which occur on a species in appropriate laboratory tests will generally occur on the same species in comparable field conditions. A numerical WQC can be considered as the highest concentration of a certain substance that would not cause any unacceptable long-term or short-term effect on the aquatic organisms or their use. Because aquatic ecosystems can tolerate some stress and occasional adverse effects, it is not necessary to protect all species at all times and places. Therefore, the purpose of deriving numerical national WQC is not to provide the same concentration at any time for the survival and the reproduction of all species in a specific ecosystem, but to provide adequate protection to ecologically and commercially important species in waters at most times, and to avoid overprotection or underproduction [9].

## 4. Conclusions

This study has shown that among the four metals tested on freshwater organisms, Pb was the most toxic, followed by Fe, Ni, and Zn. The CMC and CCC values for Fe, Pb, Ni, and Zn estimated from this study are 37.2, 8.5, 82.5, and 152.4 *μ*g L^−1^, and 9.0, 2.0, 19.9, and 36.7 *μ*g L^−1^, respectively. These values are suggested to be used in Malaysian WQC for metals in freshwater ecosystems for the protection of aquatic life.

## Figures and Tables

**Table 1:** Acute toxicity of Fe, Pb, Ni, and Zn to eight freshwater species (mean with 95% confidence limits).

Metal	Rank	Species	96 h-LC_50_ (mg L^−1^)
	1	*N. elinguis *	0.12 (0.06–0.17)
	2	*S. major*	0.28 (n.a)
	3	*D. melanostictus*	0.4 (n.a)
Iron	4	*C. javanus*	0.62 (0.004–1.25)
	5	*P. reticulata *	1.46 (0.47–2.57)
	6	*R. sumatrana*	1.71 (0.035–4.46)
	7	*M. lanchesteri *	3.42 (0.35–32)
	8	*M. tuberculata*	8.49 (1.58–15.25)

	1	*M. lanchesteri*	0.035 (0.024–0.051)
	2	*S. major*	0.53 (0.31–0.9)
	3	*N. elinguis*	0.58 (n.a)
Lead	4	*R. sumatrana*	0.63 (0.22–1.77)
	5	*C. javanus*	0.72 (0.34–1.16)
	6	*D. melanostictus*	1.5 (0.4–2.2)
	7	*P. reticulata*	1.99 (0.69–4.14)
	8	*M. tuberculata*	6.82 (2.89–12.67)

	1	*N. elinguis*	0.64 (0.56–0.69)
	2	*R. sumatrana*	0.83 (0.30–1.56)
	3	*C. javanus*	5.32 (2.79–9.21)
Nickel	4	*M. lanchesteri*	8.1 (2.1–37.3)
	5	*M. tuberculata*	8.46 (3.53–14.02)
	6	*D. melanostictus*	8.8 (5–17)
	7	*P. reticulata*	15.62 (10.77–20.56)
	8	*S. major*	19.74 (14.77–26.38)

	1	*R. sumatrana*	0.46 (0.23–0.89)
	2	*M. lanchesteri*	0.52 (0.33–0.88)
	3	*N. elinguis*	0.91 (0.79–1.13)
Zinc	4	*P. reticulata*	1.05 (0.37–2.15)
	5	*S. major*	1.19 (0.95–1.48)
	6	*M. tuberculata*	3.9 (1.81–6.67)
	7	*D. melanostictus*	4.2 (2.1–7.5)
	8	*C. javanus*	5.57 (3.54–29.42)

n.a—not available: values could not be calculated from probit software.

**Table 2:** FAV and CMC value for Fe, Pb, Ni, and Zn.

	Fe (*μ*g L^−1^)	Pb (*μ*g L^−1^)	Ni (*μ*g L^−1^)	Zn (*μ*g L^−1^)
FAV	74.5	17.0	165.0	304.9
CMC = 1/2 FAV	37.2	8.5	82.5	152.4
CCC = FAV/ACR^∗^	9.0	2.0	19.9	36.7

^∗^ACR = 8.3 (from Raimondo et al. [19]).

**Table 3:** Comparison criteria of metal concentration in freshwater ecosystem.

	Fe (*μ*g L^−1^)	Pb (*μ*g L^−1^)	Ni (*μ*g L^−1^)	Zn (*μ*g L^−1^)
This study	CMC = 37.2	CMC = 8.5	CMC = 82.5	CMC = 152.4
	CCC = 9.0	CCC = 2.0	CCC = 19.9	CCC = 36.7
USEPA	n.a.	CMC = 10.8^∗^	CMC = 120^∗^	CMC = 30^∗^
		CCC = 0.42^∗^	CCC = 13.3^∗^	CCC = 30^∗^
CCME	300	1	25	30
UNECE (Class I)	n.a.	<0.1	<15	<45
NWQS (Class II)	1000	50	50	5000

Sources: CCME [34]; NWQS [2]; USEPA [32]; UNECE [33].

n.a.—not available.

^∗^For water hardness 20 mg L^−1^.
